# Autologous Blood Patch for Persistent Ascites Leak from Non-Closing Paracentesis Tracts

**DOI:** 10.3390/medsci7090088

**Published:** 2019-08-22

**Authors:** Nazia Khan, Kevin M. Dushay

**Affiliations:** 1 Warren Alpert Medical School, Brown University, Rhode Island Hospital, Internal Medicine, 593 Eddy St, Providence, RI 02903, USA; 2 Warren Alpert Medical School, Brown University, Rhode Island Hospital, Pulmonary, Critical Care & Sleep Disorders 593 Eddy St, Providence, RI 02903, USA

**Keywords:** ascites leak, paracentesis, ascites, blood patch, cirrhosis, liver failure, complications

## Abstract

Ascites, the fluid accumulation in the peritoneal cavity, is most commonly seen in patients with end-stage liver disease (ESLD). Evaluating ascites or providing symptomatic relief for patients is accomplished by performing a paracentesis. Ascites leak from a paracentesis site can be a complication of the procedure and is associated with increased morbidity. Currently, the best options for these patients include medical management or surgical abdominal wall layer closure. Utilizing a blood patch provides an alternative approach to managing such patients. A two-center prospective case series was performed evaluating the efficacy of the blood patch in patients with significant persistent ascites leak following a paracentesis. About 30 mL of the patients’ peripheral blood was used for the blood patch. Subjects were recruited over a period of one year and followed for 30 days after the procedure. A total of six patients were recruited for this study. Subjects underwent placement of autologous blood patch at the site of the ascites leak and 100% had resolution of the leak within 24 h. None of the subjects developed any complications of the procedure. This study shows that an autologous blood patch is an effective, low-risk treatment method for ascites leaks following a paracentesis. It is a simple bedside procedure that can reduce morbidity in patients with end-stage liver disease.

## 1. Introduction

Ascites is the accumulation of fluid within the peritoneal cavity. Though there are numerous etiologies of ascites, the most common cause in the United States, accounting for approximately 80% of cases, is cirrhosis [1]. The evaluation of ascites for possible causes and assessment for spontaneous bacterial pneumonitis requires fluid sampling by abdominal paracentesis. Patients with end-stage liver disease (ESLD) with diuretic-resistant ascites may require frequent therapeutic paracenteses. One complication of this simple bedside procedure is a 5% risk of ascitic fluid leak [2], as seen in a study by De Gottardi et al. in patients with hepatic ascites undergoing large-volume therapeutic paracentesis [2]. Leaks can occur if a Z-tract has not been properly performed during the procedure, a large skin incision has been made, or if a large-bore needle is used. Though leaks can be medically managed, patients with ESLD may develop persistent significant drainage from non-closing paracentesis tracts. Significant morbidity is associated with protracted leaks, including hepatic failure and a high risk of infection [3]. Current management of ascitic fluid leak includes the medical optimization of ascites with diuretic therapy, repeat therapeutic paracentesis with proper technique, or placing an ostomy bag over the site until drainage ceases. In cases of persistent drainage, surgical closure of abdominal wall layers is standard [4], though there is a higher likelihood of complications from abdominal surgery in cirrhotic patients. Complete drainage of ascites by laparoscopically placed drains have also been used with better results than open procedures [3]. In a pilot study by Sadik et al., 14 ESLD patients with persistent ascites leak (10 due to ruptured hernia and four post-paracentesis) were treated with fibrin glue injection with favorable outcomes [4]. Fibrin sealants are made from donor plasma or cryoprecipitate and have documented adverse reactions, including hypotension, anaphylaxis, and potential transmission of an infectious disease.

Alternatively, autologous blood patches have widely been used in settings of persistent cerebral spinal fluid (CSF) leaks following a lumbar puncture, persistent air leaks in pneumothorax, as well as post-amniocentesis amniorrhea [5]. No studies have been performed that demonstrate the use of a blood patch in non-closing paracentesis sites. We conducted a study of patients with persistent significant drainage from non-closing paracentesis tracts and their outcomes following experimental treatment with an autologous blood patch.

## 2. Methods

An Institutional Review Board (IRB)-approved, two-center prospective case series was performed. Patients with persistent drainage from non-closing paracentesis tracts were recruited for one year from the inpatient setting at Rhode Island Hospital and The Miriam Hospital in Providence, Rhode Island. Subjects were included in the study if they had ascites diagnosed on physical exam or radiographic imaging, presence of an ascites leak from a recent therapeutic or diagnostic paracentesis site with failure of improvement with conservative management, and persistence of leakage for three or more consecutive days. Patients were excluded if there was presence of an overlying skin infection, bacteremia, severe coagulopathy (international normalized ratio or INR > 3) or thrombocytopenia (platelets < 20,000/mL), or ascitic leak secondary to other etiologies aside from non-closing paracentesis tract, such as umbilical hernia rupture, leaking trocar sites, or abdominal surgical site. An IRB-approved consent form was reviewed with included subjects prior to the procedure. Patients were followed for response and clinical status immediately post-procedure, as well as at 24 h, 7 days, and 30 days following the intervention. Subjects were monitored for risks including site infection, allergic reaction, ascitic fluid leakage from blood patch site, and peritonitis.

### Technique

**Figure 1:**
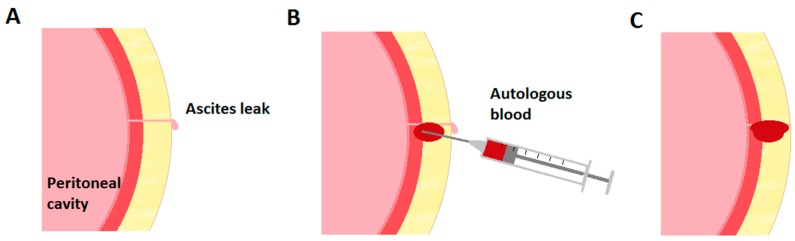
Performing an autologous blood patch. (**A**) Ascites leak is present following a paracentesis that did not utilize the Z-track technique. (**B**) Autologous blood is injected adjacent to the site of ascites leakage. (**C**) Iatrogenic hematoma obliterates the paracentesis tract.

The procedure was performed at the bedside under sterile technique with sterile gloves, masks, and antiseptic solution, as described by Thomsen et al. regarding the performance of paracentesis [6]. Approximately 30 milliliters (mL) of peripheral venous blood was drawn from the patient’s arm. The blood was immediately injected adjacent to the leaking paracentesis tract in the subcutaneous tissue and deeper into the peri-leak tissue to create an iatrogenic hematoma to obliterate the tract (Figure 1). Injection was continued while withdrawing the needle from the tissue to ensure seal of the needle puncture site. The procedure site was subsequently covered by a sterile dressing.

## 3. Results

### 3.1. Study Population

A total of six patients who met the inclusion and exclusion criteria were recruited for this study. The subjects were all male with a mean age of 58.7 ± 10.7 years (Table 1). Most had alcoholic cirrhosis and an average body mass index (BMI) of 35.9 ± 8.3 kg/m^2^. The average number of days of ascites leakage was 6 ± 1 with the amount of fluid leak ranging from 50 to 1900 mL per day.

### 3.2. Blood Patch Outcomes

**Figure 2:**
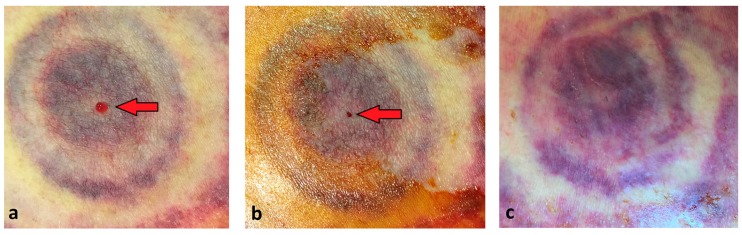
Ascites leak after autologous blood injection. (**a**) Leakage of ascitic fluid as indicated by the arrow. (**b**) Autologous blood has been injected and ascites leak has stopped immediately following procedure. (**c**) No recurrence of leak noted at 24 h.

All six subjects underwent the placement of an autologous blood patch. Due to issues with peripheral venous access, the amount of venous blood used for the patch in two patients was 17–18 mL. The remainder of subjects received a 30 mL blood patch. Only one patient experienced ascites leakage immediately following the placement of the blood patch (Table 2). All patients had complete resolution of ascites leakage after 24 h without recurrence upon follow-up at days 7 and 30 (Figure 2). None of the patients developed adverse effects or complications from the procedure.

## 4. Discussion

Ascites leak following either therapeutic or diagnostic paracentesis may resolve spontaneously with conservative management. However, autologous blood patch should be considered in patients with persistent significant leakage. The current standard of treatment is medical management. In ESLD patients with significant ascites leak, including from paracentesis sites and ruptured umbilical hernias, surgical closure of the abdominal wall layers becomes necessary [4]. However, surgery in cirrhotic patients is associated with a high risk of complications. Using the fibrin glue or cyanoacrylate glue technique has also been described [3,4,7], though there is also a risk of adverse reactions to components of the fibrin glue. Using the patient’s peripheral blood as a blood patch minimizes the risk of adverse reactions. In this study, no side effects of the procedure were noted in any of the patients.

This study also demonstrated the excellent efficacy of a blood patch for a persistent ascites leak. In this study, treatment with a blood patch resulted in 100% resolution of ascites leak by 24 h in all subjects. One patient had persistent leakage immediately following the procedure; however, this resolved at 24 h.

The use of autologous blood patch for post-procedural leaks have been described in the setting of CSF leaks following lumbar punctures and spinal anesthesia as well as persistent air leaks in pneumothoraces. There are several underlying mechanisms that may explain the effect of a blood patch. Injection of blood adjacent to the leak causes the displacement of volume and compression of the subcutaneous tissues [8,9,10]. This may explain the immediate resolution of the ascites leakage. The patch may also function as a fibrin plug [8,10], which may subsequently seal the abdominal wall hole. These mechanisms have also been described for the epidural blood patch.

The recommended blood volume in an epidural blood patch is controversial and has ranged from 2 to 20 mL [8,11]. In pneumothoraces with persistent air leak, a volume of 50 mL of blood has been utilized to attain pleurodesis [12,13]. In our study, all subjects except two received 30 mL of blood for the patch. Among the two, one patient had persistent ascites leakage immediately after receiving 18 mL of blood volume for the patch, which subsequently resolved at 24 h. This may have been due to a combination of the subject’s high BMI and lower blood volume in the blood patch. Therefore, depending upon patient characteristics, 20–30 mL of peripheral blood may be an appropriate volume for a paracentesis leak. The volume of blood required may depend on multiple factors, such as the size of the tract, the degree of coagulopathy, indices affecting the intra-abdominal pressure, and factors affecting wound healing in this patient population. However, further studies need to be performed to assess this particular aspect of the intervention.

This is a preliminary study to suggest the feasibility and advantages of utilizing a blood patch for persistent ascites leak. Although ongoing medical management may have eventually resulted in resolution of the ascitic leaks, each patient was referred for this procedure after several days of persistent leak despite conservative treatment. In addition, medical management is a significantly more expensive approach to this problem since a protracted leak can lead to prolonged hospital stays. Furthermore, persistent fluid collection or a damp dressing against the skin places the patient at risk for skin breakdown at the site. This procedure reduces patient risks and discomfort.

Limitations of this study include a small sample size consisting entirely of males. This is likely due to the rarity of ascites leaks seen in the inpatient setting. A larger sample size could be possible in outpatient settings where ESLD patients requiring repeated paracenteses are seen more frequently.

This study demonstrated that the blood patch for persistent leaks is cost-effective with a high efficacy and safety profile. Additional studies are needed to further refine the intervention, such as the assessment of appropriate blood volume. Though controlled trials could be performed, they would not be blinded and would serve only to document how much longer the fluid leak persists in those treated medically. Studies may also be considered to compare the blood patch with a normal saline injection as a control group, which may help further elucidate the underlying mechanism and efficacy of a blood patch, or alternative therapeutic options. A prospective trial studying epidural blood patch with epidural saline infusion did show effectiveness with saline, though there was reduced efficacy in the saline group when compared to the blood patch [14].

## Figures and Tables

**Table 1:** Baseline characteristics of subjects.

Characteristic *	Study Subjects (*n* = 6)
Age	58.67 ± 10.7
Male (%)	6 (100)
Race (%)	
White	4 (66)
Hispanic	1 (17)
Asian	1 (17)
Etiology of cirrhosis (%)	
Alcohol	3 (50)
Hepatitis C	2 (33)
Drug-induced	1 (17)
Coronary artery disease (%)	0 (0)
Body mass index (BMI) in kg/m^2^	35.90 ± 8.30
Creatinine in mg/dL	1.00 ± 0.43
INR in s	1.58 ± 0.33
Platelets ×10^9^/L	104.50 ± 47
Hemoglobin in g/dL	9.70 ± 1.87
Paracentesis (%)	
Diagnostic	2 (33.3)
Therapeutic	4 (66.7)
Ascites fluid removed at paracentesis in mL (range)	3306 (30–6500)
Ascites fluid leak in mL/day (range)	630 (50–1900)
Number of days leaking since paracentesis	6 ± 1

* Values are expressed as the means ± standard deviation.

**Table 2:** Blood patch outcomes.

Time Post-Procedure	Immediate	24 h	7 Days	30 Days
Subjects with fluid leakage post-procedure (*n* = 6)	1 *	0	0	0

* In this subject, 18 mL of peripheral venous blood was used.
